# National health data linkage and the agreement between self-reports and medical records for middle-aged and older adults in Taiwan

**DOI:** 10.1186/s12913-018-3738-x

**Published:** 2018-12-03

**Authors:** Ching-Ju Chiu, Hsiang-Min Huang, Tsung-Hsueh Lu, Ying-Wei Wang

**Affiliations:** 1 Institute of Gerontology, College of Medicine, National Cheng Kung University, No. 1, University Road, Tainan, Taiwan 70101; 2 Institute of Public Health, College of Medicine, National Cheng Kung University, Tainan, Taiwan; 3 Health Promotion Administration, Ministry of Health and Welfare, Taipei, Taiwan; 4 School of Medicine, Tzu Chi University, Hualien, Taiwan

**Keywords:** Demography, Epidemiology, International issues, Measurement, Methodology, Self-rated health, Linked data (data linkage), Accuracy of self-reports, Elderly, Diabetes, Hypertension

## Abstract

**Background:**

Characteristics associated with acceptance of dataset linkages and health data linkage data quality were analyzed.

**Methods:**

Participants from the 2011 Taiwan Longitudinal Study on Aging were asked to link their epidemiological data with concurrent and future medical claim datasets. Characteristics associated with acceptance of data linkage, data consistency, under-reporting, and over-reporting of disease conditions were identified.

**Results:**

Among the 3727 respondents, 3601 (96.6%) accepted data linkage. Middle-aged adults with worse functional health accepted data linkage. Older adults (65+) with better health behavior and social support were more likely to accept data linkage. Consistency between self-reports and medical data was very good to satisfactory (Kappa = 0.80 and 0.67, respectively, for diabetes and hypertension). Comorbidities were common risk factors resulting in inconsistency between self-reports and medical data (OR = 1.58 and 1.27, respectively, for diabetes and hypertension). Living alone was another risk factor resulting in inconsistency for diabetes. Male, older, and not living alone were other risk factors resulting in inconsistencies for hypertension. Under-reporting of illness was associated with poor health and older age. Over-reporting of illness was associated with better health and younger age.

**Discussion:**

The findings suggest different adjustment methods for middle-aged versus older respondents when considering self-report data validity.

## Background

First-hand information and medical records can be obtained from questionnaires or interviews. However, these data tend to have specific advantages and disadvantages, and such limitations make it difficult for researchers to obtain complete information for analysis. Therefore, in recent years, epidemiological studies have not only conducted analysis on single data, but the depth and breadth of research has been improved by linkage to different types of data [1]. In addition to lessening the burdens on researchers by avoiding repeating the same tasks, data linkage can also allow acquisition of longitudinal data from respondents, so researchers will have a more complete understanding of the health conditions of respondents [2]. Nevertheless, when the Personal Information Protection Act was enacted in Taiwan in 1995, although the privacy of personal information was secured, it also made it harder to conduct exchange of different types of data since an inquiry must be conducted to ascertain if the respondent is willing to accept linkages to personal data or medical records. For example, the Taiwan Longitudinal Study on Aging (TLSA) began inquiring if respondents were willing to accept linkage of their personal data in the National Health Insurance Research Database (NHIRD) in 2007. Literature reviews have also demonstrated that sociodemographic characteristics of participants, such as age, gender, race, setting, and socioeconomic and health status, are associated with incomplete data linkage and potential for systematic bias in reported outcomes [3]. However, to our knowledge, few studies have been conducted to investigate differences in attributes between middle-aged and elderly respondents who are willing to or unwilling to accept linkage to epidemiological data and medical records.

In Taiwan, although the National Health Insurance Research Database covers medical records for more than 99% of the general public and offers abundant clinical diagnosis information, the content might vary owing to differences in accessibility to medical services and individual behavior related to the use of such services. Thus, analyses of “self-reports” is typically used in epidemiological studies. However, some studies have also suggested that self-reports are prone to deviate from reality owing to socio-demographic attributes (i.e., age, gender, level of education, comorbidities, etc.), as well as the cognitions and memories of the respondents [4]. This phenomenon has been proven to be more frequent when elderly people are interviewed [4, 5]. Therefore, there have been many nationwide studies on the accuracy of self-reports of chronic disease in foreign countries [6], and in Taiwan, there have been some studies on the accuracy of self-reports on illness among the general public. For example, Goldman et al. used biological indicators in the “Social Environment and Biomarkers of Aging Study (SEBAS)” to investigate the accuracy of self-reports on hypertension and diabetes and adopted the biomarkers collected by the first interview wave in 2000 as the diagnostic criteria. However, because the sample was small (*N* = 1003), it might not have represented the overall conditions of the entire country [7]. Wu et al. conducted the Taiwan National Health Interview Survey (NHIS) in 2005 with the National Health Insurance Research Database (NHIRD) and conducted an analysis of the accuracy of self-reports related to 14 chronic diseases and clinical diagnoses, where the sample included 15,574 research subjects over the age of 12 who were willing to accept data linkage to the National Health Insurance Research Database. However, since nearly 25.2% of the respondents in this study were unwilling to accept data linkage, and high levels of inconsistency existed between respondents who were willing and those who were unwilling to accept data linkage, over-estimation might have occurred [8].

After we reviewed the past literature on the accuracy of self-reports and medical records, we found that only a minority of studies on the quality of self-reports have involved in-depth research on relevant topics across Asia, and most of such studies have failed to further analyze and investigate the types of inconsistencies between self-reports and medical records, such as under-reporting of illness, where a specific illness exists based on medical records but it doesn’t based on self-reports. Such studies have also ignored the over-reporting of illness, where a specific illness doesn’t exist based on medical records but exits based on self-reports, as well as other relevant factors. Thus, in addition to analyzing differences in the sampled respondents in terms of who among them are willing or unwilling to accept linkages to actual medical utilization data in a large-scale demographic survey, this study also explores the differences between people with inconsistent results between the self-reports of a chronic disease and medical records, under-reporting and over-reporting of illness, and people with consistent results between the self-reports and medical records in terms of socio-demographic factors, health conditions, medical utilization, and behavioral factors among those willing to accept data linkage.

## Methods

### Sources of information

In this study, we used the Taiwan Longitudinal Study on Aging (TLSA) and the National Health Insurance Research Database (NHIRD) in 2011 to analyze the difference between self-reports and medical records among elderly people.

The Taiwan Longitudinal Study on Aging (TLSA) [9] is an on-going nationally representative survey conducted by the Population Survey and Research Center of the National Health Bureau on the health and life issues. The first wave, conducted in 1989, involves a national representative sample of adult residents comprising 4049 participants aged 60 and above in non-aboriginal townships of Taiwan. The second wave of the survey was conducted in 1993 and included interviews with 3155 survivors of the initial sample. The third wave, conducted in 1996, included 2669 survivors aged 67 and older from the 1989 survey (1047 died, and 333 dropped out in this wave, leaving 66% of the initial sample) and included 2462 nationally representative individuals aged 50 to 66, whose inclusion resulted in a replenishment of the younger part of the age distribution of respondents. As a result, since 1996, the TLSA sample is representative of the entire Taiwanese population age 50 and older living in the community and in nursing homes (*N* = 5131), and their follow-up data in 1999, 2003, 2007, and 2011.

The National Health Insurance Research Database (NHIRD) [10] was established along with National Health Insurance on March 1, 1995. As of today, more than 99% of the general public and 97% of the medical institutions and clinics in Taiwan are covered by National Health Insurance. The National Health Insurance Research Database (NHIRD) includes the basic demographic information and medical records of patients. This data, used in the current study, includes “ambulatory care expenditures by visit,” “inpatient expenditures by admission,” and “cause of death data.”

### Research subjects

The sample in 2011 from the seventh TLSA survey, which is an on-going nationally representative survey, included 3727 respondents at or over the age of 58 who had completed the interview, achieving high response rates (88.3%) [9], where a total of 3601 patients were willing to accept linkage to their NHI data, thus accounting for 96.6% of the entire population.

Regarding the differences between self-reports and medical records for diabetes and hypertension, the question “Do you have Diabetes/Hypertension?” as specified in the questionnaire for the TLSA was adopted for self-reports. As for medical records, receiving outpatient medical treatment three times or being hospitalized once for the specific disease in “the current year” were adopted as the Gold Diagnostic Criteria (the Gold Standard), for which the ICD-9 Disease Codes of Diabetes and Hypertension were 250 and 401–405, respectively.

### Research variables

#### Independent variables

The TLSA survey data was used in this study to compare the respondents willing and unwilling to accept data linkage as it related to their socio-demographic variables (gender, the age, level of education, married, or living alone), health behavior (smoking, chewing betel nuts, alcohol consumption, exercise, diet, and social support), health conditions and use of medical services. Among these, “chewing betel nuts” and “use of traditional Chinese medicine” are special behaviors unique to Taiwan. Meanwhile, we also investigated factors defined by the TLSA survey data that might result in inconsistency between self-reports and medical records, which included socio-demographic variables (gender, age, ethnicity, the level of education, and living alone) and health conditions (comorbidities, self-reported health conditions, cognitive function, and depression).

Regarding the socio-demographic attributes, ethnic groups comprised the Islanders, the Mainlanders, and the Hakka people (including other people). The level of education was divided into no education, and primary school, junior high, or higher. Health behavior in the questionnaire comprised “regular exercise,” where exercise was defined as participating in sports three times a week. In the diet section, “weight control” and “diet control” were used to judge if the respondent’s diet was healthy. If the respondent was implementing weight control and diet control, the respondent was considered to be on a healthy diet. If the respondent was implementing either weight control or diet control, the respondent was considered to be on a slightly healthy diet. If the respondent was implementing neither weight control nor diet control, the respondent was considered to be on an unhealthy diet. In the social support section [11]**,** there were five questions that concerned “whether or not there was someone to listen to you, the degree of care, satisfaction with the degree of care, whether or not there was someone to rely on when you were sick, and whether there was someone to ask your opinion” where each question contributed to the total score in a range of 0 to 20 points. The higher the score, the higher the social support. As for health conditions, the question “Do you have any diseases?” was asked in the comorbidities section, where there were 8 options, including hypertension, diabetes, heart disease, stroke, cancer, lung disease, arthritis or rheumatism, and hip fracture to choose from as the continuous variables. The higher the score, the more comorbidities there were. In the self-reported health conditions section, the question “How do you feel about your present health condition?” was asked, where there were 5 options, including “Very Good,” “Good,” “Normal,” “Not Good,” and “Very Bad” to choose from as the continuous variables. The higher the score, the better the health condition the respondents thought they had based on self-reports. In the basic activity section (Strength and Mobility Activities and Mobility), 9 questions from the Physical Mobility and Strength Scale [12] were adopted. However, the situation where the respondent remained in a sedentary position for two consecutive hours was excluded. The higher the score, the lower the mobility. In the activity of daily life (ADL) section, the modified Daily Life Assessment Scale [13] was adopted to assess the 6 questions on “Dining,” “Getting on/off a Bed or Chair,” “Dressing Oneself,” “Going to the Restroom,” “Taking a Shower,” and “Walking across a room.” The higher the score, the weaker their ADL. In the section on instrumental activities of daily living(IADL), the Instrumental Activities of Daily Living Scale [14] was adopted, and 6 questions comprising “Buying Personal Daily Necessities,” “Dealing with Money,” “Driving a Vehicle or Taking the Train Alone,” “Doing Heavy Work at Home or in the Neighborhood,” “Doing Easy Housework,” and “Making Phone Calls” were asked. The higher the score, the weaker the IADL. In the cognitive function section, 9 questions from the Short Portable Mental State Questionnaire (SPMSQ) [15] were adapted to ask questions like “Where do you live?,” “What place is this?,” “What is today’s date?,” “What day is today?,” “How old are you this year?,” “What is the surname of your mother’s original family?,” “Who are the incumbent and the previous presidents in Taiwan?” as well as some math questions. The higher the score, the stronger the cognitive ability. The answers to these questions comprised a set of continuous variables. In the depression section, 10 questions from the CES-D scale [16] were asked to investigate whether the respondent “didn’t want to eat anything, had a bad appetite, felt that it was very difficult to do everything, couldn’t sleep well, was in a bad mood, felt very lonely (without a partner), felt that people around were unfriendly, felt sad, was not motivated to do things (is not energetic), felt very happy, and felt life was good.” The two questions on whether the respondent felt happy and felt that life was good were reverse questions, where the scores of the two questions were reversed. Thus, the higher the score, the more depressed the respondent was. In the medical services utilization section, the respondents were asked, “How many times did you go to an outpatient clinic or use traditional Chinese medicine in the past year?”

#### Dependent variables

In this study, the consistency between the self-reports of chronic diseases (i.e., diabetes and hypertension) and the gold diagnostic criteria was investigated. Three possible situations were examined as follows: 1) Risk factors resulting in inconsistency between the self-reports and medical records were analyzed, where the reference group showed consistency between the self-reports and medical records, 2) risk factors resulting in under-reports of illness among the respondents were analyzed, where the reference group showed awareness of illness in both the self-reports and medical records, and 3) risk factors resulting in the over-reporting of illness (i.e. those who claimed to be ill but were actually healthy were analyzed, for which the reference group showed no illness based on both the self-reports and medical records.

### Statistical analysis

SAS 9.4 was used for data processing and analysis. First, we conducted a descriptive analysis on the sampled respondents willing and unwilling to accept linkage to the data from the National Health Insurance Research Database. An independent sample t-test was conducted on the continuous variables, while a chi-square test was conducted on the categorical variables. Then, we used testing for the Kappa coefficient to analyze the consistency between self-reports and medical records. In general, when the Kappa coefficient was larger than 0.8, there was excellent consistency; when the Kappa coefficient was between 0.6 and 0.8, there was good consistency; when the Kappa coefficient was between 0.4 and 0.6, there was mediocre consistency; when the Kappa coefficient was smaller 0.4, there was bad consistency. Finally, we used a logistic regression to investigate relevant factors resulting in inconsistencies between self-reports and medical records for diabetes and hypertension.

## Results

### Differences between respondents willing and unwilling to accept data linkage to the national health insurance research database. (Table 1)

The number of those willing to accept data linkage was 3601 (96.6%), for whom the average age was 72, and most were female (51.0%). There were no statistically significant differences found between those willing and unwilling to accept data linkage. In terms of the use of medical services, there were statistically significant differences (*p* = 0.040) between respondents willing and unwilling to accept data linkage only in terms of western-style outpatient medical treatment. Among those willing to accept data linkage, most had received a western-style outpatient medical treatment only once (41.6%), while among those unwilling to accept data linkage, most were unable to utilize western-style outpatient medical treatment (42.9%). In terms of the health behavior of the respondents, there were statistically significant differences between those willing and those unwilling to accept data linkage only in terms of smoking (*p* = 0.023) and social support (*p* = 0.008). At present, among those willing to accept data linkage, 13.1% were smokers, for whom the social support score was 9.13 ± 3.27 points, while among those unwilling to accept data linkage, 6.4% were smokers, for whom the social support score was 8.22 ± 3.33 points.

**Table 1:** Testing of differences in terms of socio-demographic variables, health conditions, medical utilization data, and health behavior among respondents willing and unwilling to accept data linkage

	Entire			< 65 (58–64)			65+		
	NO (%)	YES (%)	T/X^2^ Value	NO (%)	YES (%)	T/X^2^ Value	NO (%)	YES (%)	T/X^2^ Value
	*N* = 126 (3.38%)	*N* = 3601 (96.61%)		*N* = 45 (3.46%)	*N* = 1256 (96.54%)		*N* = 81 (3.33%)	*N* = 2345 (96.67%)	
Socio-demographic variables									
Age Mean ± SD (min-max/median)	71.26 ± 9.97 (58–93/70)	71.15 ± 9.98 (58–101/70)	0.12	60.89 ± 2.01 (58–64)/61	60.61 ± 1.96 (58–64)/61	0.95	77.02 ± 7.67 (65–93/76)	76.80 ± 7.71 (65–101/76)	0.26
Female (ref = Male)	69 (54.8)	1838 (51.0)	0.67	22 (48.9)	641 (51.0)	0.08	47 (58.0)	1197 (51.0)	1.53
Level of education			1.73			1.96			0.74
No education	19 (15.1)	684 (19.0)		0 (0.0)	37 (3.0)		19 (23.5)	647 (27.6)	
Primary school	59 (46.8)	1707 (47.4)		17 (37.8)	597 (47.5)		39 (48.1)	1095 (46.7)	
Junior high or higher	48 (38.1)	1210 (33.6)		28 (62.2)	622 (49.5)		23 (28.4)	603 (25.7)	
Married	87 (69.1)	2316 (64.3)	1.19	40 (88.9)	997 (79.4)	2.43	47 (58.0)	1319 (56.3)	0.10
Living alone	14 (11.1)	322 (8.9)	0.70	3 (6.7)	80 (6.4)	0.01	11 (13.6)	242 (10.3)	0.89
Health conditions									
Comorbidities (8 options)	1.13 ± 1.17	1.35 ± 1.21	−1.96	0.71 ± 0.90	0.93 ± 1.02	−1.43	1.37 ± 1.24	1.57 ± 1.25	−1.44
Self-reported health conditions (Number of questions = 1; total score = 5)	3.25 ± 0.99	3.17 ± 0.98	0.81	3.39 ± 0.99	3.43 ± 0.93	−0.32	3.15 ± 0.99	3.01 ± 0.98	1.17
Mobility (Number of Questions = 9; Total Score = 27)	6.33 ± 8.47	5.81 ± 7.85	0.72	1.02 ± 2.21	1.74 ± 4.20	−2.05*	9.27 ± 9.21	8.00 ± 8.46	1.33
IADL (Number of questions = 6; total score = 18)	3.45 ± 6.33	3.1 ± 5.53	0.61	0.07 ± 0.45	0.59 ± 2.51	−5.40***	5.33 ± 7.24	4.45 ± 6.20	1.25
ADL (Number of questions =6; total score = 18)	2.21 ± 5.31	1.53 ± 4.35	1.41	0.00 ± 0.00	0.26 ± 1.85	−5.00***	3.43 ± 6.31	2.21 ± 5.09	1.73
CESD (Number of questions = 10; total score = 30)	4.19 ± 5.18	4.76 ± 5.9	−0.86	2.61 ± 3.41	3.54 ± 4.74	−1.75	5.25 ± 5.77	5.34 ± 6.14	−0.12
SPMSQ (Number of questions = 9; total score = 9)	8.06 ± 1.69	8.16 ± 1.43	−0.59	8.86 ± 0.35	8.68 ± 0.69	3.21**	7.52 ± 2.01	7.84 ± 1.66	−1.28
Medical utilization									
Outpatient clinic visits			6.43*			4.87			4.03
0	54 (42.8)	1215 (33.7)		23 (51.1)	554 (44.1)		31 (38.3)	661 (28.2)	
1	39 (31.0)	1498 (41.6)		9 (20.0)	444 (35.4)		30 (37.0)	1054 (45.0)	
2+	33 (26.2)	888 (24.7)		13 (28.9)	258 (20.5)		20 (24.7)	630 (26.8)	
Traditional Chinese medicine	18 (14.3)	364 (10.1)	2.31	10 (22.2)	169 (13.5)	2.81	8 (9.9)	195 (8.3)	0.25
Health behavior									
Smoking	8 (6.4)	473 (13.1)	4.99*	3 (6.7)	232 (18.5)	4.09	5 (6.2)	241 (10.3)	1.45
Chewing betel nuts	3 (2.4)	137 (3.8)	0.68			0.12			0.73
Alcohol consumption	29 (23.0)	995 (27.6)	1.30	16 (35.6)	468 (37.3)	0.05	13 (16.1)	527 (22.5)	1.87
Exercise			1.92			0.17			4.13*
Irregular (Less than 3 times a week)	63 (50.0)	1576 (43.8)		18 (40.0)	541 (431)		45 (55.6)	1035 (44.1)	
Regular (3 times or more a week)	63 (50.0)	2025 (56.2)		27 (60.0)	715 (56.9)		36 (44.4)	1310 (55.9)	
Diet			5.06			0.17			8.44*
Unhealthy diet	43 (34.1)	1235 (34.3)		11 (24.4)	339 (27.0)		32 (39.5)	896 (38.2)	
Slightly healthy diet	54 (42.9)	1243 (34.5)		15 (33.3)	392 (31.2)		39 (48.2)	851 (36.3)	
Healthy diet	29 (23.0)	1123 (31.2)		19 (42.2)	525 (41.8)		10 (12.3)	598 (25.5)	
Social support (Number of questions = 5; total score = 20)	8.22 ± 3.33	9.13 ± 3.27	−2.64**	8.88 ± 3.20	9.27 ± 3.20	−0.77	7.74 ± 3.39	9.04 ± 3.31	−2.84**

* *p* < .05, ** *p* < .01, *** *p* < .001

Among the middle-aged adults (58–64 yrs), there were statistically significant differences between those willing and those unwilling to accept data linkage in terms of their mobility (1.74 vs 1.02, *p* = 0.045), ADL (0.26 vs 0.00, *p* < 0.001), IADL (0.59 vs 0.07, *p* < 0.001) and cognitive function (8.68 vs 8.86, *p* = 0.002), indicating that the middle-aged people willing to accept data linkage were mostly those with worse health conditions. Meanwhile, among the elderly people aged 65 or over, there were also statistically significant differences between those willing and those unwilling to accept data linkage in terms of exercise, diet, and social support, indicating that the elderly people willing to accept data linkage were mostly those with better health behavior.

### Relevant factors influencing respondents with inconsistency between self-reports and medical records, under-reports of illness, or over-reporting of illness for diabetes and hypertension

Table 2 shows that the sensitivity of the self-reports on diabetes was 82.5%; the specificity was 96.3%; the overall consistency was 93.5%, and the Kappa value was 0.80 (95%CI = 0.77–0.82). As such, the consistency between self-reports and medical records was “excellent.” Meanwhile, the sensitivity of the self-reporting on hypertension was 84.1%; the specificity was 83.3%; the overall consistency was 83.7%, and the Kappa value was 0.67 (95%CI = 0.65–0.70). As such, the consistency between self-reports and medical records was “good.”

**Table 2:** Distribution of respondents offering answers consistent and inconsistent with medical records in their self-reports on diabetes and hypertension and the test of consistency

		Self-reports					
		Diabetes			Hypertension		
		Yes	No	Total	Yes	No	Total
Medical records (Gold Standard)	Yes	597 (82.5%)	127 (17.5%)	724 (100.0%)	1416 (84.1%)	267 (15.9%)	1683 (100.0%)
	No	106 (3.7%)	2771 (96.3%)	2877 (100.0%)	320 (16.7%)	1598 (83.3%)	1918 (100.0%)
Sensitivity of self-report		82.5%			84.1%		
Specificity of self-report		96.3%			83.3%		
Overall consistency		93.5%			83.7%		
The Kappa coefficient		0.80 (0.77–0.82)			0.67 (0.65–0.70)		

Self-reports: Do you have diabetes and/or hypertension?

Medical records: Receiving outpatient medical treatment three times or more or being hospitalized once in 2011 for a specific disease. The ICD-9 Disease Codes of Diabetes and Hypertension were 250 and 401–405, respectively

Table 3 predicts relevant factors resulting in inconsistency in the self-reporting of illness, under-reports of illness (under-estimation), and the over-reporting of illness (over-estimation). First, we found that the number of comorbidities was the common risk factor resulting in inconsistency in the self-reporting of diabetes and hypertension, whereas living alone was another risk factor resulting in inconsistency in the self-reporting of diabetes, and male gender, older age, or not living alone were risk factors resulting in inconsistency in the self-reporting of hypertension. Second, we limited the size of the sample with illness based on medical records to understand the factors influencing under-reports of illness among the respondents, for which the results showed that risk factors resulting in under-reports of illness (i.e. diabetes and hypertension) include older age, poor health conditions based on self-reports, and a lower number of comorbidities. Third, we limited the size of the sample without illness based on medical records to understand the factors influencing the over-reporting of illness among the respondents (i.e. risk factors causing the respondents to claim illness when they actually were not ill), and we found that the common risk factors resulting in the over-reporting of illness (i.e. diabetes and hypertension) among the respondents include male gender and a higher number of comorbidities, while younger age was another risk factor resulting in the over-reporting of diabetes. Meanwhile, being an Islander with better health condition based on self-reports were the risk factors resulting in over-reporting of hypertension.

**Table 3:** Relevant factors influencing inconsistency in the self-report on illness, under-reports of illness (under-estimation), and over-reporting of illness (over-estimation)

	Inconsistency		Under-reports of illness		Overly reports of illness	
	Diabetes N = 3601	Hypertension N = 3601	Diabetes *N* = 724	Hypertension *N* = 1683	Diabetes *N* = 2877	Hypertension *N* = 1918
	OR (95% CI)	OR (95% CI)	OR (95% CI)	OR (95% CI)	OR (95% CI)	OR (95% CI)
Female (ref: Male)	0.86 (0.63–1.17)	0.75 (0.61–0.93)**	1.32 (0.81–2.14)	0.86 (0.61–1.22)	0.49 (0.29–0.82)**	0.59 (0.41–0.85)**
Age	1.00 (0.98–1.02)	1.02 (1.01–1.04)**	1.05 (1.02–1.08)*	1.03 (1.01–1.05)**	0.95 (0.93–0.98)**	1.00 (0.98–1.02)
Ethnic groups (ref: Mainlander)						
Islander	1.14 (0.69–1.90)	1.27 (0.89–1.81)	0.69 (0.32–1.49)	0.77 (0.45–1.30)	1.77 (0.74–4.23)	2.24 (1.1–4.59)*
Hakka people (including other people)	1.04 (0.58–1.87)	1.11 (0.74–1.66)	1.05 (0.44–2.55)	0.63 (0.34–1.17)	1.05 (0.37–2.95)	2.03 (0.94–4.40)
Level of education (ref: Junior high or higher)						
No education	0.67 (0.40–1.12)	1.02 (0.73–1.43)	0.46 (0.20–1.08)	0.87 (0.51–1.50)	1.44 (0.67–3.07)	1.67 (0.91–3.05)
Primary school	0.87 (0.62–1.21)	1.24 (0.98–1.55)	0.81 (0.49–1.36)	1.31 (0.90–1.91)	0.85 (0.49–1.46)	1.33 (0.91–1.94)
Living alone	1.62 (1.04–2.52)*	0.68 (0.47–0.98)*	2.00 (0.99–4.03)	0.67 (0.39–1.15)	1.57 (0.75–3.28)	0.88 (0.46–1.68)
Self-reported health conditions	0.88 (0.74–1.04)	1.06 (0.94–1.19)	0.73 (0.55–0.97)*	0.82 (0.68–0.99)*	0.90 (0.68–1.18)	1.61 (1.31–1.98)***
Comorbidities	1.58 (1.40–1.79)***	1.27 (1.16–1.38)***	0.29 (0.21–0.38)***	0.25 (0.20–0.31)***	3.62 (2.9–4.52)***	7.67 (6.08–9.68)***
Cognition (SPMSQ)	0.94 (0.84–1.04)	0.95 (0.89–1.03)	1.05 (0.87–1.27)	0.91 (0.81–1.01)	0.87 (0.75–1.02)	1.01 (0.87–1.16)
Depression (CESD)	0.99 (0.96–1.01)	1.02 (1.00–1.04)	0.99 (0.94–1.03)	1.02 (0.99–1.05)	0.98 (0.94–1.02)	1.01 (0.98–1.05)

* *p* < .05, ** *p* < .01, *** *p* < .001

## Discussion

This is the first study in Taiwan to examine sociodemographic, functional, behavioral, and medical utilization characteristics of survey respondents who are willing or unwilling to have their data linked with their medical claim dataset. It also unprecedentedly identified the existence of age group differences and also clarified data quality with not only agreement, but also under-reporting and over-reporting of health conditions. It was found that middle-aged adults with worse functional health and older adults with better health behavior and social support profiles were more likely to agree to data linkage. While comorbidities were common risk factors resulting in inconsistency between self-reports and medical data, living alone was another risk factor of self-report bias for diabetes status. Furthermore, inconsistency in hypertension reports were also associated with being male, older, and not living alone. Under-reporting of illness was associated with poor health and older age, and over-reporting of illness was associated better health and younger age.

Few studies have been conducted to investigate differences between respondents willing or unwilling to accept datasets linkages. Among the handful of existing studies, few have differentiated among age differences or have only used small, regional samples. A cross-sectional survey with a small sample of 228 Taiwanese aged 65 years and older was conducted between 1992 and 1993 in Taipei. It indicated that there were no statistically significant differences in the features between those unwilling to accept data linkage and those willing to accept data linkage. However, among those willing to accept data linkage, the number of people with illness based on self-reports was higher than that among those unwilling to accept data linkage (36% vs 24%, *p* < 0.01) [5]. Although the findings of that study were contradictory to those of the present study, our study may be more accurate because we used a national survey of middle aged and older people, and the survey respondents were interviewed in 2011, which may be more representative of current older adults. Another existing study using a nationally representative database on people aged 54 or over was conducted in 2000 in Taiwan [7], where a database similar to that used in this study was used. The results indicated that those willing to accept data linkage engaged more in social activities, but their ADL and self-reported health condition were weaker. Our results extend Goldman’s findings because we found that attitude toward data linkage may be very different between middle-aged and older adults, where unhealthy middle-aged adults (i.e. 58–64 y) with weaker physical functions (i.e. more limitations in Mobility, ADL, IADL and cognitive functions) were prone to accept data linkage, while elderly people aged 65 or over with regular exercise, a healthy diet, and greater social support were more likely to agree to data linkage. Another study based on a questionnaire survey for self-reports and medical records of chronic disease in Canada found that those willing to accept data linkage were mostly aged between 20 and 64 (83.9%) without comorbidities based on self-reports (65.7%) [17]. We felt that the results of the Canadian studies might differ with our results owing to age and cultural differences.

We analyzed the consistency between self-reports and medical records on diabetes and hypertension, where receiving outpatient medical treatment three times or more or being hospitalized once in the past year were used as the criteria for medical records on diabetes and hypertension. We found the Kappa values for diabetes and hypertension to be 0.80 (showing an excellent consistency) and 0.66 (showing good consistency), respectively. Then, we further loosened the criteria, so that receiving outpatient medical treatment three times or being hospitalized once in the past year were used to calculate the accuracy of self-reports on illness, but the Kappa values were not improved (the data are not displayed). The results of past studies using the data retrieved from Taiwan [5, 7, 8] corresponded to the results of this study, and the consistency regarding diabetes was better than that regarding hypertension. Based on a systematic review of the past literature, we found that if the diagnostic criteria for diabetes (ICD-9 Disease Code: 250) remain the same, and the same self-reports questionnaire was adopted, the Kappa value regarding diabetes ranged between 0.79 and 0.81 [18]. The Kappa value obtained in this study was right within that range. Meanwhile, in this study, the consistency regarding hypertension was better than that in a study conducted by Goldman et al. [7], where the Kappa value was 0.41. This might be because the biological indicators used for clinical diagnosis were limited to a current blood sample. If the respondent had hypertension and controlled the illness with medicine to make the blood pressure fall within the normal range, we could not accurately judge if the sampled respondent had hypertension.

This study further investigated whether different socio-demographic variables and the health or cognitive conditions of the respondents would influence the consistency between the self-reports and the medical records. The result showed that male respondents could have engaged in over-reporting of illness (i.e. diabetes and hypertension), which caused the inconsistency between the self-reports and the medical records illness on hypertension. This phenomenon might be related to the different medical behavior of males and females [19], where the results of this study were consistent with those of other studies [4]. Older age is often associated with under-reports of illness (i.e. diabetes and hypertension) as well as inconsistencies between self-reports and the medical records related to hypertension, which is consistent with certain arguments proposed in some of the past literature [7, 8]. Thus, we might infer that older people might contract multiple diseases that could damage their memory functions, so they cannot clearly remember their own diseases. This study also found that self-reports in younger people often over-estimated diabetes, which was consistent with the results obtained by SC et al., although there were no statistically significant differences in the relevant data [5]. Meanwhile, this study also found that Islanders engaged in over-reporting of hypertension, while living alone acted as a risk factor causing inconsistency regarding diabetes but a protection factor for hypertension, which should be further clarified in the future.

Regarding the health conditions of the respondents, the number of comorbidities and self-reported health conditions were important factors causing differences between the self-reports and medical records. When the number of comorbidities was high, this resulted in inconsistency regarding diabetes and hypertension and also over-reporting of illness, which was consistent with the findings of other studies [4]. This may be because the respondents could have been confused when they had multiple diseases. When the number of comorbidities is lower, there may be under-reports of illness based on self-reports among patients with diabetes and hypertension. A study was conducted to investigate over-estimation (the over-reporting of illness) and under-estimation (under-reports of illness based on self-reports) of cardiovascular diseases, hypertension, and diabetes based on self-reports [5], for which the results also showed that when the number of comorbidities was lower, under-estimation of diabetes and hypertension tended to occur, which was consistent with the results of this study. Meanwhile, under-reports of illness (i.e. diabetes and hypertension) based on self-reports tended to occur among respondents whose self-reported health conditions were poor. This might be because respondents whose self-reported health conditions were poor might conceal their pre-existing diseases or neglect their own health, so they do not know about their pre-existing diseases. Nevertheless, it was interesting that respondents whose self-reported health conditions were better instead engaged in over-reporting of hypertension. This phenomenon showed that elderly people often over-report hypertension because hypertension is a common chronic disease among elderly people.

Several limitations for this study should be considered. First, respondent’s willingness may be influenced by the length of the time linkages, so we can’t be sure that the results will be the same if the linkage time is increased or decreased. Second, our results for data consistency, under-reports, and over-reports of health conditions only focus on two major chronic diseases, diabetes and hypertension. Thus, we can’t be sure as to whether these predictive factors are representative of all disease conditions for middle-aged and elderly people that are self-reported, and we are still unclear whether other diseases have different predictive factors for under-reports or over-reports. This should be explored further in future research.

To summarize, it was found that acceptance of data linkage among middle-aged adults (58–64 yrs) occurred to a greater degree in those with worse functional health, but older adults (65+) who were more likely to accept data linkage comprised those with better health behavior and social support. While comorbidities were common risk factors resulting in inconsistency between self-reports and medical data, living alone was another risk factor for self-report bias for diabetes status. Furthermore, inconsistencies in hypertension reports were also associated with being male, older, and not living alone. Older respondents or those with poor health were more prone to under-reporting of illness, while those who were younger or who had better health were prone to over-reporting of illness. The findings from this study suggest different adjustment methods for middle-aged versus older respondents when considering the validity of self-reported data. In addition, because groups with over-reporting of illness might abuse medical resources, and groups with under-reporting might neglect their health, the findings from this study also provide information regarding identification of risk groups, so policies can be developed to address medical resource abuse or health neglect.

## Conclusions & Implications

This study using two nationally representative datasets linkage showed good consistency between self-reports and medical records regarding common chronic diseases (i.e. hypertension and diabetes) among older Taiwanese adults. However, middle-aged and elderly people showed different health or behavioral characteristics in terms of their willingness to link epidemiological data with medical records: middle-aged adults (58–64 yrs) exhibited worse functional health to a greater degree, but older adults (65+) who were more likely to accept data linkage comprised those with better health behavior and social support. Based on the state-of-the-art missing data treatment recommendations [20–22], incorporating auxiliary variables that have high correlations (at least > .5 to ensure sizable effects) with dependent variables [23], through either multiple imputation (MI) or maximum-likelihood (ML) methods, will improve the efficiency of data validity. The variables identified in the present study shown to have high associations with willingness of data linkage variables, such as outpatient clinic visits, smoking and social support, especially the willingness of middle-aged adults (58–64 yrs) were influenced by functional health, and the willingness of older adults (65+) was influenced by health behavior and social support. These variables were suggested to be good auxiliary variables for linked data or incomplete data when conducting imputation.

## Abbreviations

ADL: Activity of daily life
CES-D scale: Center for Epidemiologic Studies Depression Scale
IADL: Instrumental activities of daily living
MI: Multiple imputation
ML: Maximum-likelihood
NHI: National Health Insurance
SPMSQ: Short Portable Mental State Questionnaire
TLSA: Taiwan Longitudinal Study on Aging
